# Next-Generation Sequencing for Molecular Diagnosis of Cystic Fibrosis in a Brazilian Cohort

**DOI:** 10.1155/2021/9812074

**Published:** 2021-02-03

**Authors:** Amanda Cambraia, Mario Campos Junior, Verônica Marques Zembrzuski, Ricardo Magrani Junqueira, Pedro Hernán Cabello, Giselda Maria Kalil de Cabello

**Affiliations:** ^1^Human Genetics Laboratory, Oswaldo Cruz Institute, Oswaldo Cruz Foundation, Rio de Janeiro, Brazil; ^2^Molecular and Cellular Biology Postgraduate Program, Federal University of the State of Rio de Janeiro, Rio de Janeiro, Brazil; ^3^Laboratory of Bacterial Zoonoses, Oswaldo Cruz Institute, Oswaldo Cruz Foundation, Rio de Janeiro, Brazil; ^4^Laboratory of Genetics, School of Health Science, University of Grande Rio, Rio de Janeiro, Brazil

## Abstract

Cystic fibrosis (CF), an autosomal recessive genetic disease, is recognized as one of the most prevalent diseases in Caucasian populations. Epidemiological data show that the incidence of CF varies between countries and ethnic groups in the same region. CF occurs due to pathogenic variants in the gene encoding cystic fibrosis transmembrane conductance regulator (*CFTR*), located on chromosome 7q31.2. To date, more than 2,000 variants have been registered in the *CFTR* database. The study of these variants leads to the diagnosis and the possibility of a specific treatment for each patient through precision medicine. In this study, complete screening of *CFTR* was performed through next-generation sequencing (NGS) to gain insight into the variants circulating in the population of Rio de Janeiro and to provide patient access to treatment through genotype-specific therapies. Samples from 93 patients with an inconclusive molecular diagnosis were subjected to full-length screening of *CFTR* using an Illumina NGS HiSeq platform. Among these patients, 46 had two pathogenic variants, whereas 12 had only one *CFTR* variant. Twenty-four variants were not part of our routine screening. Of these 24 variants, V938Gfs∗37 had not been described in the CF databases previously. This research achieved a molecular diagnosis of the patients with CF and identification of possible molecular candidates for genotype-specific treatments.

## 1. Introduction

The cystic fibrosis transmembrane conductance regulator gene (*CFTR*; OMIM #602421) encodes a chloride channel that is located in the apical membrane of epithelial cells [1]. Variants in this gene cause a reduction or complete absence of channel activity, leading to the development of a life-threatening illness known as cystic fibrosis (CF; OMIM #219700) or mucoviscidosis [2]. CF is characterized as a multisystem disease with an autosomal recessive inheritance pattern. Patients exhibit progressive manifestations of obstructive pulmonary disease, pancreatic insufficiency, and high concentrations of chloride in the sweat [3–5].

With the identification of *CFTR* in 1989 [6], genetic analysis to identify disease-causing variants in this gene began, improving the diagnosis of CF and identification of pathogenic variant carriers. The most prevalent pathogenic variant was discovered 30 years ago, having a deletion of a phenylalanine at position 508 of the protein (F508del; c.1521_1523delCTT; p.Phe508del), present in one or both alleles in approximately 90% of cases in some populations [7, 8]. Additionally, genetic studies helped clarify the correlation between CFTR dysfunction and the clinical characteristics, revealing that defects in *CFTR* can create other phenotypes besides CF [9–11].

Currently, more than 2,000 variants have been described over all 27 exons of *CFTR* (http://www.genet.sickkids.on.ca/cftr/StatisticsPage.html), although only some of them are pathogenic [12]. Pathogenic variants are grouped into six classes according to their primary biological defects [13]. Understanding the process of CFTR synthesis up to its targeting to the plasma membrane is essential for the development of specific treatments. These treatments could be used to correct defective CFTRs according to the pathogenic variant of each patient. The genotype-specific therapeutic approach focuses on the detection of small modulatory molecules capable of correcting deficient subcellular trafficking of CFTR (“correctors”) or on the defective gating (“potentiators”) [14, 15]. The identification of pathogenic variants is important for early diagnosis, allowing a more effective treatment and a longer life expectancy for the patients [12, 16].

The heterogeneous distribution of *CFTR* variants worldwide and the size of the gene represent major challenges for the molecular diagnosis of CF. Thus, establishing population-specific mutation panels is extremely important [17, 18]. With new sequencing technologies becoming easily available, it is possible to rapidly generate a large amount of sequencing data, expanding the analysis of *CFTR* and uncovering population-specific mutation panels, increasing the sensitivity and specificity of available diagnostic strategies for various populations or ethnic groups [19, 20].

The aim of this study was to perform a complete screening of *CFTR* through next-generation sequencing (NGS) to investigate the variants prevalent in the population in Rio de Janeiro, Brazil.

## 2. Materials and Methods

### 2.1. Ethics Statement

The study protocol was approved by the Ethics and Research Committee of the Oswaldo Cruz Foundation (CAEE: 55095316.4.0000.5248/Protocol No: 2.010.565/17). All participants provided written informed consent prior to their inclusion in this study.

### 2.2. Patients and Samples

**Figure 1:**
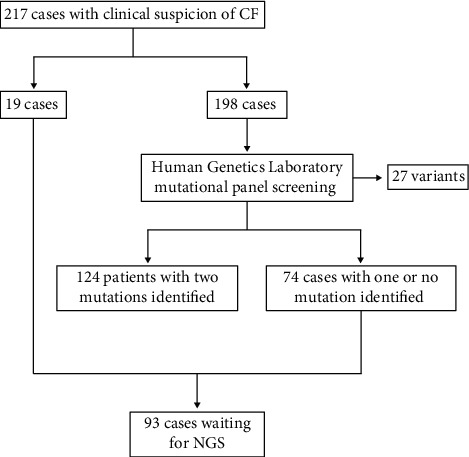
Flowchart of the studied sample. Of 198 probands with clinical suspicion of cystic fibrosis (CF), 74 candidates for next-generation sequencing (NGS) were part of an initial genetic screening of 27 variants. After the first stage, these patients were found to have one or no mutated *CFTR* allele. Additionally, 19 patients had not undergone any initial screening, totaling 93 patients.

Patients from three specialized centers, namely, the Pulmonology Sector of the National Institute of Women and Adolescent Health Fernandes Figueira/FIOCRUZ, the Pedro Ernesto University Hospital/UERJ, and the Carioca Association of Assistance to Mucoviscidosis, were received in the Human Genetics Laboratory of the Oswaldo Cruz Institute for molecular diagnosis of CF. All individuals were undergoing treatment and were recruited by specialized clinicians. Patients with clinical manifestations suggestive of CF, i.e., positive sweat test (>60 mEq), positive newborn screening test, or suggestive clinical features following the most recent diagnosis of CF guidelines [21], were invited to participate in this study. In total, 217 patients agreed to participate in the study (Figure 1). Most of these patients (198 individuals) were initially screened for 27 known CF variants (Table 1) through our routine molecular panel test, and both mutated alleles were found in 124 patients. In the remaining 74 patients, only one mutated allele (36 patients) or no mutated alleles (38 patients) were found. Nineteen patients were not tested in this initial panel. To search for the missing pathogenic variants in these 93 patients, we sequenced the entire *CFTR* (exons and introns) through NGS.

The 93 unrelated patients comprised 44 males and 49 females (age range, 4–47 years). In addition, two control subjects were included: a healthy control and a carrier control with the 3849+10KBC>T (c.3717+12191C>T) intronic variant. Additionally, a reaction control from the NGS kit was used.

### 2.3. DNA Extraction

Genomic DNA was extracted from peripheral blood leukocytes using a PureLink Genomic DNA Kit (Invitrogen, Carlsbad, CA), according to the manufacturer's instructions.

### 2.4. NGS

For sequencing, we used an Illumina HighSeq System (Illumina, USA) from the IOC High-Performance Sequencing Platform. Targeting of the region of interest from the entire sequence of *CFTR*, containing exons and introns, was achieved by designing a custom enrichment kit using the Illumina Design Studio tool. A total of 990 amplicons were generated to cover 158,462 nucleotides from the entire *CFTR*, from nucleotide chr7:117,119,917 to chr7:117,308,801 (188,884 bases), resulting in 83.89% of coverage. *CFTR* exon coordinates were obtained from the human genome assembly Hg19 (Genome Reference Consortium).

Each sample was quantified using a Qubit® 2.0 fluorometer (Applied Biosystems, Life Technologies Corporation, Carlsbad, CA). For library preparation and enrichment of the targeted regions, we utilized the TruSeq® Custom Amplicon kit (Illumina Inc., San Diego, CA, USA), following the standard protocol. The final enriched library was sequenced via HiSeq 2500 (Illumina Inc., San Diego, CA, USA) using 150 bp paired-end reads.

Data were processed as follows: reads were trimmed using Trimmomatic (v.0.35) [22] for the removal of Truseq adapters and low-quality bases. Afterward, the reads were mapped against the reference sequence of the 990 targeted amplicons using the BWA-MEM (Burrows-Wheeler Aligner) version 0.7.17 algorithm [23]. The sequence alignment map (SAM) file was sorted and converted using samtools-1.3 [24]. Variants were called using freebayes (v.1.1.0) [25]. At the end of the process, variants were sorted and annotated using SnpSift (v.4.3) [26] and SnpEff (v.4.3) [27] and compared with the available databases dbSNP (https://www.ncbi.nlm.nih.gov/SNP/), ClinVar (https://www.ncbi.nlm.nih.gov/clinvar/), and Cystic Fibrosis Mutation Database (CFMDB; http://www.genet.sickkids.on.ca/app).

### 2.5. Sanger Sequencing

Twenty-four variants detected through NGS were confirmed by Sanger sequencing. Fifteen variants were previously observed in our diagnostic routine panel either by Sanger sequencing performed previously or through restriction fragment length polymorphism (RFLP). As a result, all variants observed through NGS were confirmed by at least one additional method. Sanger sequencing was performed using a Big Dye Terminator V3.1 kit (Applied Biosystems, Austin, TX, USA) in an ABI PRISM 3130xl DNA analyzer (Applied Biosystems). The *CFTR* DNA amplification was achieved with the set of primers listed in Supplementary Table 1. PCR products were visualized on 1.5% agarose gels and purified using a Sweep Clean up kit (Applied Biosystems, Vilnius, Lithuania). The obtained sequences were aligned with the reference sequence of *CFTR* in Ensembl (ENST00000003084.10). Sequence analysis was performed with Chromas Lite 2.0 software (Technelysium) and BioEdit Sequence Alignment Editor v6.0.6 (Ibis Therapeutics).

## 3. Results

Of the 309,361,463 pairs of reads generated in our experiment, 97.52% passed the quality control, of which 99.73% were assigned individually to each of the 95 subjects, with an average of 6,364,353 reads per individual. The lowest number and highest number of reads in the samples were 1,593,382 and 17,301,187, respectively. On an average, 88.73% of the reads were successfully mapped with the reference, indicating the quality of the data. The mean reading coverage over the region of interest was 4,000 times, with a standard deviation of 2,445, indicating a satisfactory depth. The minimum base quality parameters of 20 and a minimum mapping quality of 30 were used for detecting the variants. These results indicate high resolution and high capacity for variant identification.

Analysis of the NGS data allowed us to identify 39 variants (Table 2); 24 were not part of our routine screening. Of these, 22 were exonic and 2 were intronic; 14 already had their confirmed pathogenicity in the CFTR2 database (https://www.cftr2.org/). The 24 variants were found in exons 3, 4, 6, 8, 10, 12, 13, 14, 15, 17, 19, 20, and 22 and in introns 5 and 19, totaling 13 missense, 6 nonsense, 2 frameshift, 1 deletion, and 2 splicing variants.

A new frameshift variant, V938Gfs∗37 (c.2812_2813insG; p.Val938GlyfsX37), which occurs in exon 17 of *CFTR*, where guanine is inserted at position 2812 of the cDNA, resulting in a stop codon 37 bases after the amino acid change, was reported. This variant was identified in heterozygosity with the F508del mutation in a 27-year-old male patient with a positive sweat test (>60 mEq) and typical CF respiratory manifestations. *In silico* analyses by MutationTaster showed that V938Gfs∗37 is predicted as pathogenic and affects the structure of CFTR. The variant was submitted to the CFMDB.

Based on these results, both mutated alleles were identified in 46 individuals, 12 individuals presented only one CF variant, and 35 presented no genetic variant related to CF. Among the patients with two variants identified, 37 had the genotype CF-causing/CF-causing, 9 patients presented the combination CF-causing/unknown clinical significance, and 1 had CF-causing/novel variant, according to the CFTR2 database.

## 4. Discussion

The identification of new variants causing CF continues to occur even after almost 30 years of *CFTR* identification. Currently, more than 2,000 variants have been registered in the CFMDB; however, only 442 are annotated in the CFTR2 database, of which 360 are considered pathogenic. These variants vary in frequency and distribution in different populations. Historically, CF has been regarded as a disease limited to people of European descent. However, research has shown that CF is not ancestry linked. Therefore, in order to obtain a high detection rate, diagnosis through population-specific mutation panels should consider the molecular heterogeneity of the population and the variants to be included [11, 19]. For example, panels used in European populations to diagnose African descent patients eventually lead to inconclusive results [28]. In countries with heterogeneous populations, such as those in Latin America, the use of these panels also leads to misdiagnosis, which can compromise the patient's health and treatment [29–32]. In Brazil, with its highly mixed population, the choice of mutation panels designed for other populations has become ineffective for the diagnosis, leading to a low detection rate [33]. This shows the importance of NGS for diagnostics in these populations.

Genetic testing of CF in Brazil is not performed with uniformity, since there are no epidemiological studies or a comprehensive neonatal screening to estimate the incidence of the disease in different regions of the country. Raskin [34] estimated that only 10% of the patients are diagnosed, leading to a false impression of low incidence in the Brazilian population. According to the latest report of the Brazilian Registry of Cystic Fibrosis (REBRAFC), a large increase was observed in the percentage of patients with genotype investigation. In 2013, 40.6% of the patients in a total of 2,942 individuals had their genotyping performed, and in 2017, the number of patients genotyped reached almost 80% of the 5,128 individuals analyzed. This improvement is due to advances in molecular diagnostic techniques [35].

Here, we used NGS in a cohort of 93 patients to conclude their molecular diagnosis and search for new or rare CF pathogenic variants in the Brazilian population. Thus, 74 patients from a sample of 198 individuals were tested. These individuals had already been screened for 27 common CF pathogenic variants. This means that patients with both alleles identified from this panel were not used in this study, causing the frequency of these common pathogenic variants to be underestimated by our NGS results.

The four most frequent pathogenic variants observed in our sample of 217 patients were F508del in 42% alleles, wherein 72 patients were heterozygous, and 55 were homozygous for this pathogenic variant. The 3120+1G>A, G542X, and G85E variants were observed in 5.8%, 4.1%, and 3.2% alleles, respectively. All four variants were part of the routine testing for CF molecular diagnosis performed in our laboratory. Nunes et al. [36] published the first Brazilian study using a NGS methodology using Ion Torrent PGM (Life Technologies), with pediatric patients from the Children's Institute at Hospital das Clínicas of the University of São Paulo Medical School (HCFMUSP). The three most frequent pathogenic variants described in their study were F508del (59.1%), G542X (7.3%), and 3120+1G>A (5.3%). Our findings corroborate the observations presented by Nunes et al. [36], in which they justify the high frequency of the G542X variant as a result of the migration flow of Spanish, Portuguese, and Italians to Brazil between the 19^th^ and 20^th^ centuries.

The Brazilian population has a significant genetic heterogeneity, mainly resulting from a trihybrid ethnic mixture of Europeans, Africans, and indigenous populations, which varies proportionally between the different Brazilian regions [37]. Thus, it is clear that CF genetic tests preestablished for populations defined as Caucasian may present limitations when employed in a scenario as heterogeneous as the Brazilian one. For example, if we had used a panel of 23 variants recommended by the American College of Medical Genetics and the American College of Obstetricians and Gynecologists [38] that detected 88% of non-Hispanic Caucasians, we would have reduced our rate of pathogenic variant recognition. Furthermore, of the 39 variants found in our study, only 10 would be part of this panel. In addition, five patients would remain without any identified *CFTR* variant. Thus, genetic diagnosis for the 23 patients would not have been completed with variants identified in both alleles. Rispoli et al. proposed a panel of 11 variants as a complement to the screening of the F508del variant performed by the Brazilian Public Health System in Rio Grande do Sul, Brazil [39]. Despite being a panel developed for a Brazilian region, it does not include all the variants observed in our sample.

A total of 46 out of 93 patients who participated in the NGS had two pathogenic variants. Among these variants, we detected a new one, i.e., V938Gfs∗37. Predictive analysis of the possible effect of this insertion in the MutationTaster program was positive for pathogenicity. As a class I mutation, it leads to complete or near-complete loss of CFTR activity [12, 40]. Even with complete sequencing of *CFTR*, 12 patients were identified with only one *CFTR* variant. Among these, seven patients presented a defined pathogenic allele (F508del/unknown, G542X/unknown, 3120+1G>A/unknown, R334W/unknown, and 2183delAA/unknown). Notably, our designed method of sequencing is not capable of detecting large exonic and intronic deletions and duplications or copy number variations, a type of variant that is known to cause CF in some cases. We believe that such pathogenic variants may be responsible for some of these cases with only one defined allele.

Five individuals had one *CFTR* variant classified as “non-CF causing” according to CFTR2: R668C (c.2002C>T; p.Arg668Cys), G576A (c.1727G>C; p.Gly576Ala), R75Q (c.224G>A; p.Arg75Gln), and L997F (c.2991G>C; p.Leu997Phe). Two of the five patients presented the R668C and G576A variants. In 1992, Fanen et al. [41] considered the R668C variant as a polymorphism, and in 2003, Pagani et al. [42] described G576A as a variant that likely induced the skipping of exon 12 in splicing, leading to reduced levels of normal *CFTR* transcripts [43]. In a study by Ziętkiewicz et al. [44], the R668C variant was considered pathogenic and G576A a compound allele element. Based on previous studies, these variants when combined with a CF pathogenic variant are associated with a moderate phenotype (CFTR-related disorders; CFTR-RDs), in particular with congenital bilateral absence of the vas deferens (CBAVD). However, we cannot affirm that both variants R668C and G576A form a complex allele in our patients, since a segregation study could not be performed. According to El-Seedy et al., the variants G576A and R668C affect the chloride channel activity [45]. The R75Q variant leads to the exchange of arginine to glutamine. Zielenski et al. [46] initially reported R75Q as a neutral variant that was not involved in CF. Gené et al. [47] evaluated the impact of this variant on the functioning of the CFTR channel and found a pattern of glycosylation and subcellular distribution similar to that of wild-type CFTR. The variant L997F was initially considered as a polymorphism and was subsequently reported to cause CFTR-RDs, such as lung diseases, disseminated bronchiectasis, idiopathic pancreatitis, CBAVD, and neonatal hypertrypsinemia with normal sweat test [48].

It is now known that the severity of CF is influenced not only by *CFTR* variants but also by modifier genes, intragenic polymorphisms, environmental factors, and lifestyle, which explains individuals with the same variant having different clinical manifestations [19]. To this end, great efforts have been made to develop therapeutics for correcting the consequences of *CFTR* variants on the function of the protein [49], some of which are already available for the treatment of patients with certain genotypes [15].

## 5. Conclusions

Through the NGS-based study of *CFTR*, we expanded our knowledge of the variants that circulate in the population of Rio de Janeiro, allowing us to offer genetic support for patients seeking specific treatments. In addition, NGS made it possible to increase our previous panel of variants from 27 to 51 (41 *CFTR* pathogenic variants). This study highlights the importance of considering the distribution of pathogenic variants specific for admixed populations for choosing the right molecular diagnostic method. The use of NGS for the entire gene has an advantage over the mutation-specific panels available, allowing the discovery of disease-causing variants that are population specific. Moreover, this method provides an opportunity for patients from countries with heterogeneous populations, which are not well covered by commercial diagnostic panels, to have a molecular diagnosis for receiving genotype-specific therapy and creates the scope for providing genetic counseling to the family.

## Figures and Tables

**Table 1:** Mutation panel used in routine testing in the Human Genetics Laboratory of the Oswaldo Cruz Institute.

No	Legacy name	cDNA HGVS name	Protein HGVS name	dbSNP ID	Method^1^
1	F508del	c.1521_1523delCTT	p.Phe508del	rs113993960	Sanger sequencing
2	3120+1G>A	c.2988+1G>A	No protein	rs75096551	Real-time PCR
3	G542X	c.1624G>T	p.Gly542X	rs113993959	Real-time PCR
4	G85E	c.254G>A	p.Gly85Glu	rs75961395	Real-time PCR
5	R334W	c.1000C>T	p.Arg334Trp	rs121909011	Real-time PCR
6	3849+10KBC>T	c.3717+12191C>T	—	rs75039782	Real-time PCR
7	S4X	c.11C>A	p.Ser4X	rs397508173	Sanger sequencing
8	R1162X	c.3484C>T	p.Arg1162X	rs74767530	Sanger sequencing
9	N1303K	c.3909C>G	p.Asn1303Lys	rs80034486	Real-time PCR
10	P205S	c.613C>T	p.Pro205Ser	rs121908803	Real-time PCR
11	S549R	c.1547T>G	p.Ser549Arg	rs121909005	Sanger sequencing
12	Y1092X	c.3276C>A	p.Tyr10292X	rs121908761	Real-time PCR
13	R347P	c.1040G>C	p.Arg347Pro	rs77932196	Sanger sequencing
14	232del18	c.100_117delTTGTCAGACATATACCAA	p.Leu34_Gln39del	rs397508141	Sanger sequencing
15	R553X	c.1657C>T	p.Arg553X	rs74597325	Real-time PCR
16	W1282X	c.3846G>A	p.Trp1282X	rs77010898	Real-time PCR
17	2183del AA>G	c.2051_2052delAAinsG		rs121908799	Sanger sequencing
18	G551D	c.1652G>A	p.Gly551Asp	rs75527207	Sanger sequencing
19	2184delA	c.2052delA	p.Lys684AsnfsX38	rs121908746	Sanger sequencing
20	S549N-I	c.1646G>A or c.1646G>T	p.Ser549Asn or p.Ser549Ile	rs121908755	Sanger sequencing
21	120del23	—	—	—	Sanger sequencing
22	S168L	c.635C>T	p.Ser168Leu	rs869249241	Sanger sequencing
23	S1255X	c.3764C>A	p.Ser1255X	rs76649725	Real-time PCR
24	G1244E	c.3731G>A	p.Gly1244Glu	rs267606723	Sanger sequencing
25	CFTRdup2-3	—	—	—	MLPA^2^
26	Del25-26	—	—	—	MLPA
27	del25-27CORTBP2	—	—	—	MLPA

^1^Methodology previously used to screening mutations at the Human Genetics Laboratory of the Oswaldo Cruz Institute. ^2^Multiplex ligation-dependent probe amplification.

**Table 2:** *CFTR* variants identified through next-generation sequencing.

No	Legacy name	cDNA HGVS name	Protein HGVS name	Exon/intron	dbSNP ID	Classification^1^	Mutation panel^2^	Alleles (in 108 alleles)	Mutation type
01	R75Q^4^	c.224G>A	p.Arg75Gln	Exon 3	rs1800076	Non-CF-causing	No	1	Missense
02	Q98X^4^	c.292C>T	p.Gln98X	Exon 4	rs397508461	CF-causing	No	1	Nonsense
03	R117H^4^	c.350G>A	p.Arg117His	Exon 4	rs78655421	Varying clinical consequence	No	1	Missense
04	I148T^4^	c.443T>C	p.Ile148Thr	Exon 4	rs35516286	Non-CF-causing	No	1	Missense
05	711+1G>T^4^	c.579+1G>T	—	Intron 5	rs77188391	CF-causing	Yes	1	Intronic
06	L206W^4^	c.617T>G	p.Leu206Trp	Exon 6	rs121908752	CF-causing	No	2	Missense
07	R347H^4^	c.1040G>A	p.Arg347His	Exon 8	rs77932196	CF-causing	No	1	Missense
08	S434X^4^	c.1301C>A	p.Ser434X	Exon 10	rs367934560	CF-causing	No	1	Nonsense
09	A559T^4^	c.1675G>A	p.Ala559Thr	Exon 12	rs75549581	CF-causing	No	2	Missense
10	A561E^4^	c.1682C>A	p.Ala561Glu	Exon 13	rs121909047	CF-causing	No	1	Missense
11	G576A^4^	c.1727G>C	p.Gly576Ala	Exon 13	rs1800098	Non-CF-causing	No	2	Missense
12	S589N^4^	c.1766G>A	p.Ser589Asn	Exon 13	rs397508300	—	No	2	Missense
13	R764X^4^	c.2290C>T	p.Arg764X	Exon 14	rs121908810	CF-causing	No	1	Nonsense
14	E831X^4^	c.2491G>T	p.Glu831X	Exon 15	rs397508387	CF-causing	No	1	Nonsense
15	Y913X^4^	c.2739T>A	p.Tyr913X	Exon 17	rs149790377	CF-causing	No	2	Nonsense
16	V938Gfs∗37^4,5^	c.2812_2813insG	p.Val938GlyfsX37	Exon 17	Novel	—	No	1	Frameshift
17	L997F^4^	c.2991G>C	p.Leu997Phe	Exon 19	rs1800111	Non-CF-causing	No	1	Missense
18	3199del^l4^	c.3067_3072delATAGTG	—	Exon 19	rs121908767	—	No	1	Deletion
19	3272-26A>G^4^	c.3140-26A>G	—	Intron 19	rs76151804	CF-causing	No	2	Intronic
20	R1066C^4^	c.3196C>T	p.Arg1066Cys	Exon 20	rs78194216	CF-causing	No	4	Missense
21	W1089X^4^	c.3266G>A	p.Trp1089X	Exon 20	rs78802634	CF-causing	No	2	Nonsense
22	W1098C^4^	c.3294G>T	p.Trp1098Cys	Exon 20	rs397508533	CF-causing	No	1	Frameshift
23	Q1100P^4^	c.3299A>C	p.Gln1100Pro	Exon 20	rs397508535	—	No	1	Missense
24	I1234V^4^	c.3700A>G	p.Ile1234Val	Exon 22	rs75389940	CF-causing	No	1	Missense
25	232del18	c.100_117delTTGTCAGACATATACCAA	p.Leu34_Gln39del	Exon 2	rs397508141	—	No	1	Deletion
26	G85E	c.254G>A	p.Gly85Glu	Exon 3	rs75961395	CF-causing	Yes	7	Missense
27	S168L	c.635C>T	p.Ser168Leu	Exon 5	rs869249241	—	No	1	Missense
28	P205S	c.613C>T	p.Pro205Ser	Exon 6	rs121908803	CF-causing	No	1	Missense
29	R334W	c.1000C>T	p.Arg334Trp	Exon 8	rs121909011	CF-causing	Yes	2	Missense
30	F508del	c.1521_1523delCTT	p.Phe508del	Exon 11	rs113993960	CF-causing	Yes	29	Deletion
31	S549R	c.1547T>G	p.Ser549Arg	Exon 12	rs121909005	CF-causing	No	1	Missense
32	G542X	c.1624G>T	p.Gly542X	Exon 12	rs113993959	CF-causing	Yes	9	Nonsense
33	R668C	c.2002C>T	p.Arg668Cys	Exon 14	rs1800100	Non-CF-causing	No	3	Missense
34	2183delAA>G	c.2051_2052delAAinsG	p.Lys684SerfsX38	Exon 14	rs121908799	CF-causing	No	2	Frameshift
35	3120+1G>A	c.2988+1G>A	—	Intron 18	rs75096551	CF-causing	Yes	12	Intronic
36	Y1092X	c.3276C>A	p.Tyr10292X	Exon 20	rs121908761	CF-causing	No	2	Nonsense
37	R1162X	c.3484C>T	p.Arg1162X	Exon 22	rs74767530	CF-causing	Yes	1	Nonsense
38	3849+10KBC>T	c.3717+12191C>T	—	Intron 22	rs75039782	CF-causing	Yes	2	Intronic
39	N1303K	c.3909C>G	p.Asn1303Lys	Exon 24	rs80034486	CF-causing	Yes	1	Missense

^1^Classification based on CFTR2 database (clinical and functional translation of CFTR). ^2^23 ACMG/ACOG mutation panel—American College of Medical Genetics and the American College of Obstetricians and Gynecologists. ^3^Number of CFTR alleles found in this study. ^4^Twenty-four mutations found in the next-generation sequencing performed in this study. ^5^Novel mutation found in the next-generation sequencing performed in this study.

## Data Availability

The data generated by the next-generation sequencing are available upon request.

## Supplementary Materials

Supplementary Materials Supplementary Table 1: primers used for Sanger sequencing.
