# Supplementary material for: Next-Generation Sequencing for Molecular Diagnosis of Cystic Fibrosis in a Brazilian Cohort
Source: Dis Markers. 2021 Feb 3;2021:9812074. doi: 10.1155/2021/9812074 (PMC7878085; doi:10.1155/2021/9812074)
Supplement: Supplementary Materials — Supplementary Table 1: primers used for Sanger sequencing. [file 9812074.f1.docx]

Supplementary Table 1: Primers used for Sanger sequencing

| Exon/Intron | | Forward | Reverse |
| --- | --- | --- | --- |
| Exon 4 | GTTTCACATATGGTATGACCCTCTA | | TCCCTTACTTGTACCAGCTCAC |
| Intron 5 | AACTCCGCCTTTCCAGTTGT | | ATTTCTGCCTAGATGCTGGG |
| Exon 6 | AGGGGTGGAAGATACAATGACA | | CGCCTCTAATCCCAGCTACT |
| Exon 10 | GGGGAATTATTTGAGAAAGCAA | | TCCAAAAATACCTTCCAGCACT |
| Exon 12 | CAACTGTGGTTAAAGCAATAGTGT | | GCACAGATTCTGAGTAACCATACT |
| Exon 13 | TCAGTGAATCGATGTGGTGAC | | TTCTGCCATACCAACAATGG |
| Exon 14 | AACTGAGAGACCCCGAGGAT | | GGGAGTCTTTTGCACAATGG |
| Exon 15 | TGTATACATCCCCAAACTATC | | GGTGGCATGAAACTGTACTGT |
| Exon 17 | GTGCATGCTCTTCTAATGCA | | AAGGCACATGCCTCTGTGCA |
| Exon 20 | CAAAGAATGGCACCAGTGTGA | | TGGAAATTCAAAGAAATCACTTGTTCA |
| Intron 19 | CAAAGAATGGCACCAGTGTGA | | TGGAAATTCAAAGAAATCACTTGTTCA |
| Exon 22 | CATTGAAAAGCCCGACAAAT | | TGCTTCAGGCTACTGGGATT |
